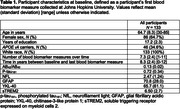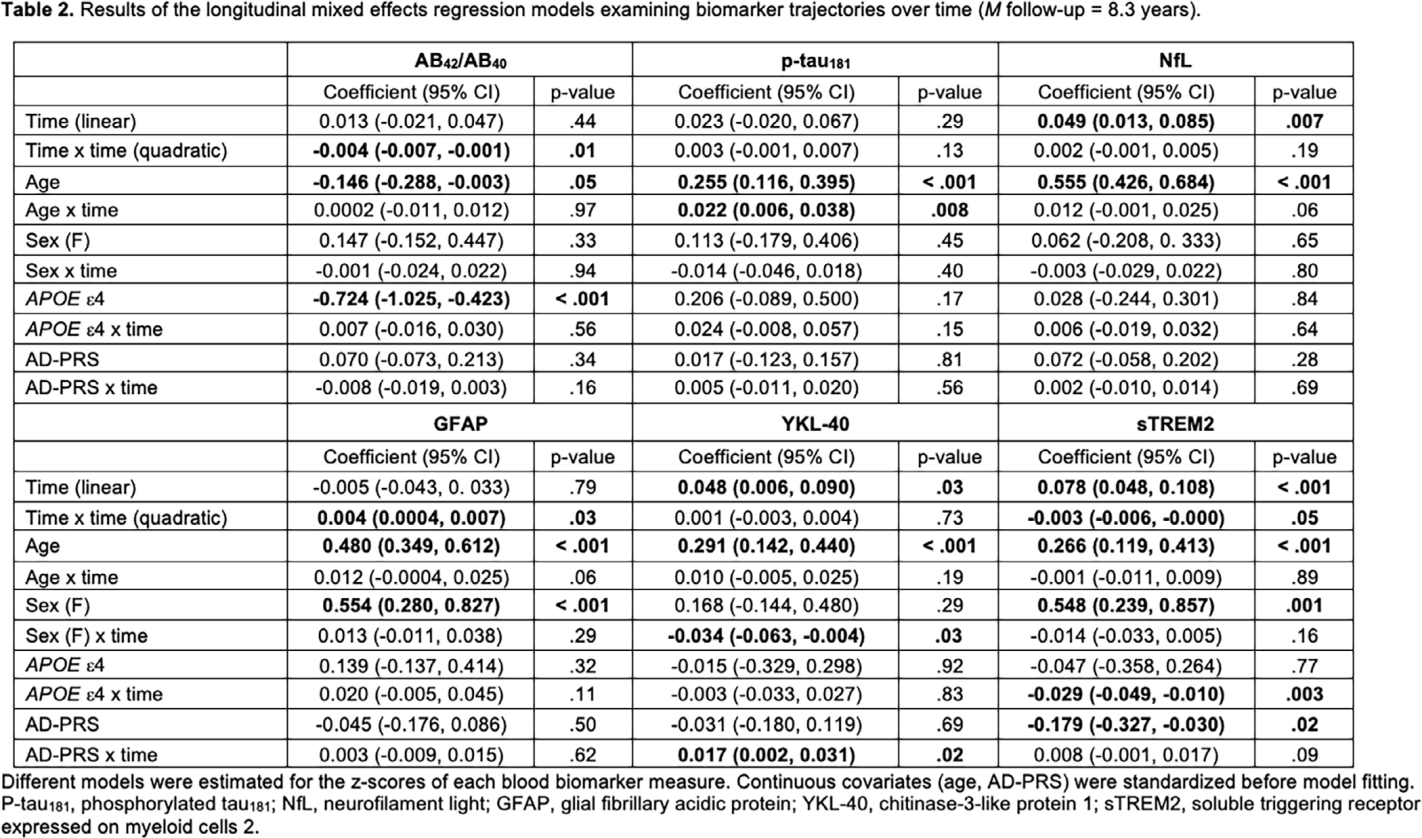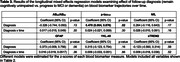# Plasma biomarker trajectories: Relationship to AD genetic risk factors among cognitively unimpaired individuals

**DOI:** 10.1002/alz.090753

**Published:** 2025-01-09

**Authors:** Corinne Pettigrew, Jiangxia Wang, Anja Soldan, Timothy J. Hohman, Logan C. Dumitrescu, Mei‐Cheng Wang, Marilyn S. Albert, Kaj Blennow, Tobias Bittner, Abhay Moghekar

**Affiliations:** ^1^ Johns Hopkins University School of Medicine, Baltimore, MD USA; ^2^ Johns Hopkins University, Baltimore, MD USA; ^3^ Vanderbilt University Medical Center, Nashville, TN USA; ^4^ Johns Hopkins Bloomberg School of Public Health, Baltimore, MD USA; ^5^ University of Gothenburg, Mölndal Sweden; ^6^ Roche Pharmaceuticals, Basel Switzerland

## Abstract

**Background:**

Few studies have examined how a range of potential modifiers may influence the trajectories of Alzheimer’s disease (AD) blood biomarkers in those who were cognitively unimpaired when first assessed. This study examined potential modifiers of longitudinal changes in plasma biomarkers, including genetic factors (i.e., APOE e4 allele and AD‐polygenic risk scores (PRS), demographics (age and sex), and follow‐up diagnoses (i.e., progression to MCI or dementia) among participants who were cognitively unimpaired at baseline.

**Method:**

Analyses included a subset of BIOCARD Study participants who had all of the relevant measures of interest, including AD‐PRS (N = 133, M baseline age = 64.7y; 7.3 biomarker measures over 8.3 years of follow‐up; Table 1); 19 (14%) had progressed to MCI or dementia over time. These analyses focused on log‐transformed and standardized values of AB_42_/AB_40_, p‐tau_181_, NfL, GFAP, YKL‐40, and sTREM2 in plasma, measured using cobas Elecsys assays in the NeuroToolKit panel (Roche Diagnostics).

**Result:**

In longitudinal mixed‐effects models, NfL, GFAP, YKL‐40, and TREM2 increased over time, whereas AB_42_/AB_40_ decreased over time (Table 2). YKL‐40 increases were greater among individuals with higher AD‐PRS and rates of sTREM2 increase were attenuated among APOE e4 carriers. Additionally, baseline AB_42_/AB_40_ levels were lower among APOE e4 carriers and baseline sTREM2 levels were lower in individuals with higher AD‐PRS. Older participants had more abnormal baseline biomarker levels, as well as greater rates of change in p‐tau_181_. Baseline GFAP and sTREM2 levels were higher among females, and rates of change in YKL‐40 were greater among males. In a separate set of models, with AD genetic risk factors covaried, those who progressed to MCI/dementia had more abnormal ptau_181_ at baseline (Table 3) compared to those who remained cognitively unimpaired, but biomarker rates of change did not differ by follow‐up diagnosis.

**Conclusion:**

Among initially cognitively unimpaired individuals, blood biomarkers were differentially related to APOE e4 genetic risk and AD‐PRS. Only YKL‐40 and sTREM2, each markers of neuroinflammation, demonstrated relationships with an AD‐PRS. Additional work is underway to explore whether lower sTREM2 values among cognitively unimpaired participants with higher AD genetic risk reflect reduced microglial activation during preclinical AD.